# SIRT7 Regulates the Vascular Smooth Muscle Cells Proliferation and Migration via Wnt/*β*-Catenin Signaling Pathway

**DOI:** 10.1155/2018/4769596

**Published:** 2018-12-04

**Authors:** Jianghua Zheng, Kai Chen, Haifei Wang, Zhilong Chen, Yong Xi, Hongshun Yin, Kun Lai, Yujuan Liu

**Affiliations:** ^1^Department of Vascular Surgery, Affiliated Hospital of North Sichuan Medical College, China; ^2^Department of Gynaecology and Obstetrics, Affiliated Hospital of North Sichuan Medical College, China

## Abstract

A huge amount of evidence indicates that sirtuin 7 (SIRT7), a key mediator of many cellular activities, plays a crucial role in the pathogenesis of various diseases. However, little is known about the role of SIRT7 in atherosclerosis. This study investigated the potential role of SIRT7 in regulating the proliferation and migration of human vascular smooth muscle cells (HAVSMCs) and its possible molecular mechanism. In this study, human vascular smooth muscle cells (HAVSMCs) were induced by oxidized low-density lipoprotein (ox-LDL) to establish atherosclerosis (AS) cell model. Immunofluorescence staining and Western blot were used to detect the level of *α*-SMA expression, which was a marker protein in AS. In addition, RT-qPCR and Western blot assay were applied for exploring the mRNA and protein expression levels of SIRT7, Wnt, *β*-catenin, and cyclin D1 after knockdown or overexpression of SIRT7. And, furthermore, Cell Counting Kit-8 assay, flow cytometry, and wound-healing assay were used to assess HAVSMCs proliferation, cell cycle, and migration. Dickkopf-1 (DKK-1), a secretory glycoprotein that can block Wnt/*β*-catenin pathway, was used in SIRT7 overexpression HAVSMCs; subsequently cells proliferation and migration were assessed by Cell Counting Kit-8 assay, flow cytometry analysis, and wound-healing assay. We found that knockdown of SIRT7 significantly promoted cell proliferation and migration, decreased the percentages of cells in the G1 and G2 phases, and increased those in the S phase and downregulated the protein expression levels of Wnt, *β*-catenin, and cyclin D1, while overexpression of SIRT7 had reverse results. After treatment with Wnt/beta-catenin pathway inhibitor DKK-1 in SIRT7 overexpression HAVSMCs, cell proliferation and migration were increased, respectively. In conclusion, SIRT7 inhibited HAVSMCs proliferation and migration via enhancing Wnt/*β*-catenin activation, which provided a novel therapeutic strategy for antiatherosclerosis.

## 1. Introduction

Atherosclerosis (AS) is a chronic degenerative disease of the arterial wall, which is well known for its high morbidity and mortality in aged people around the world [1]. A growing body of evidences have demonstrated that abnormal proliferation and migration of vascular smooth muscle cells (VSMCs) play a significant role in AS progression [2, 3]. Accumulating reports suggest that oxidized low-density lipoprotein (ox-LDL), a key risk atherogenic factor in the development progression of AS, contributes to VSMCs proliferation and migration [4, 5]. Therefore, we choose ox-LDL-stimulated VSMCs to induce AS cell model in order to explore the relative regulatory mechanisms.

The sirtuins (SIRTs) protein family belongs to class III of histone deacetylases and consists of seven members (denoted SIRT1–7). Increasing amounts of evidence indicate that SIRTs can regulate plenty of cellular processes, such as stress responses, metabolism, cell cycle, inflammation, and senescence as well as apoptosis [6]. Furthermore, SIRTs also participate and have diverse roles in cardiovascular, neurodegenerative diseases and cancer [7]. SIRT1, the most widely studied mammalian sirtuin, could suppress CD40 expression via NF-kB pathway in human umbilical vein endothelial cell [8]. It has been well documented that SIRT1 protects against DNA damage and inhibits atherosclerosis in VSMCs [9]. In addition, impaired SIRT1 promotes the migration of vascular smooth muscle cell-derived foam cells [10]. SIRT7, the latest characterized SIRT, is the youngest member in SIRTs protein family, and several compelling breakthrough reports show that SITR7 plays a decisive role in cell proliferation, migration, survival, and protein synthesis as an important cellular regulator [11–13]. The previous studies reported that SIRT7 inhibited proliferation and migration in several cancer cells [14, 15]. SIRT7 as a tumor suppressor plays an important role in suppressing the epithelial-to-mesenchymal transition in oral squamous cell carcinoma [16]. Moreover, the expression of SIRT7 was downregulated in thyroid carcinoma and lower SIRT7 levels were observed in big tumor size [17]. However, the role of SIRT7 in AS remains poorly elucidated.

A recent study shows that SIRT7 is instrumental for osteogenic differentiation of bone marrow stem cells, partly by activation of the Wnt/*β*-catenin signaling pathway [18]. Accumulating evidence shows that Wnt/*β*-catenin signaling exerts a critical role in vascular pathologies. For example, several studies suggest that the *β*-catenin/T-cell factor (TCF) signaling pathway can regulate VSMCs proliferation and vascular calcification [19, 20]. In addition, evidence is emerging to indicate that Wnt/*β*-catenin signaling participates in regulating cellular proliferation, inflammation, and cell deposition fate, which are important causes in AS progression [21–23]. All of these studies arouse our interests in whether SIRT7 and Wnt/*β*-catenin signaling play roles in resisting AS and the underlying regulatory relationship between them. Therefore, Dickkopf1 (DKK-1), a secretory glycoprotein that can block the Wnt/*β*-catenin pathway by competitively binding to receptors on the cell membrane, was used in our present study [24].

In this study, we demonstrated that the expression of SIRT7 was downregulated in HAVSMCs stimulated by ox-LDL. Moreover, SIRT7 knockdown enhances HAVSMCs proliferation and migration accompanied with decreasing protein expression levels of Wnt, *β*-catenin, and cyclin D1, while SIRT7 overexpression had opposite results in parallel studies. The protein expression levels of Wnt, *β*-catenin, and cyclin D1 were decreased after DKK-1 blocking Wnt/beta-catenin pathway. In conclusion, SIRT7 inhibited VSMCs proliferation and migration via activating Wnt/*β*-catenin.

## 2. Materials and Methods

### 2.1. Cell Culture and Exposure to ox-LDL

The human aortic vascular smooth muscle cells T/G HAVSMCs (HAVSMCs) were obtained from China Center for Type Culture Collection (Wuhan, China) and maintained in DMEM medium (Gibco, USA) supplemented with 10% fetal bovine serum (FBS, Gibco, USA), 100 *μ*g/ml penicillin, and 100 *μ*g/ml streptomycin at 37°C in a 5% CO_2_ humidified incubator. HAVSMCs were seeded in 6-well plates at the density of 1 × 10^5^ cells/well. Subsequently, they were exposed to 100 *μ*g/ml ox-LDL (Yiyuan Biotech, Guangzhou, China) for 24 h to establish the AS cell model. Cells with passage number between 10 and 15 were used for the experiments.

### 2.2. Cell Transfection

HAVSMCs were seeded in 6-well plates at the density of 1 × 10^6^ cells/well. When grown at 40–60% confluence, HAVSMCs were transfected with 100 nM of SIRT7 siRNA or control siRNA in Opti-MEM with Lipofectamine RNAiMAX (Invitrogen, Carlsbad, CA). SIRT7 siRNA (siSIRT7-1: 5′-CCUGCCGUGUGAGGCGGAA-3′ and siSIRT7-2: 5′-GCCGUGUGAGGCGGAAGCG-3′) and control siRNA (siControl: 5′-CCUUGCGUGGGAGCGCGAA-3′) were synthesized by Invitrogen (Carlsbad, CA, USA). The overexpression of SIRT7 was made with the pcDNA3.1 plasmid (BioVector, Beijing, China) which was performed using Lipofectamine 2000 (Invitrogen, Carlsbad, CA). Then successful transfections were determined by RT-qPCR and Western blot assay after cells were incubated for 24 h.

### 2.3. Immunofluorescence Staining and Analysis

HAVSMCs were seeded in 24-well plates which contained coverslips. After different treatment, cells were fixed with 4% paraformaldehyde for 15 min. Then 0.1% Triton X-100 in TBS was used to permeabilize the fixed cells for 20 min at room temperature. After being blocked in 5% bovine serum albumin for 30 min, cells were incubated with anti-*α*-SMA (Santa Cruz Biotechnology, CA, USA) overnight at 4°C. Following washing three times with PBS, cells were incubated with DyLight™ 488-conjugated secondary antibodies (Thermo Scientific) for 1 h at room temperature. Then the nuclei were subsequently stained with 4′,6-diamidino-2-phenylindole (DAPI; Sigma-Aldrich; 1:1000) for 5 min and then washed three times with PBS in the dark. Immunofluorescence was detected using a confocal laser scanning microscope (Olympus FV1000).

### 2.4. Cell Proliferation Assay

To explore the effect of SIRT7 knockdown or SIRT7 overexpression on the proliferation of ox-LDL-induced HAVSMCs, cells were seeded in 96-well plates at a density of 5 × 10^3^ cells/well (three replications per group) and incubated at 37°C for 6 h. The cell viability was examined by CCK-8 kit (Dojindo Laboratories, Kumamoto, Japan) according to the manufacturer's instruction. At the indicated time points (24, 48, and 72 h), 10 *μ*l CCK-8 solution was added to each well. Following incubation at 37°C for 1 h, the absorbance was measured with a plate reader at 450 nm.

### 2.5. Cell Migration Assay

For the wound-healing assay, cells were plated in 12-well plates at the density of 1 × 10^5^ cells/well (three replications per group). When cells were grown at 80% confluence and merged into a monolayer, a sterile 200 *μ*l pipette tip was used to gently generate scratch wounds at the surface of the cell layer. Then cells were rinsed twice with serum-free media to remove cell debris. An inverted microscope (BX51; Olympus Corporation; 20X magnification) was used to monitor cells at the borders of the scratches. The degree of scratch healing was observed and images were captured in each group at 0 and 24 h.

### 2.6. Flow Cytometry

The cell cycle analysis was performed using the cell cycle analysis kit (Beyotime Biotechnology, Jiangsu, China). Following transfection for 48 h, 1 × 10^5^ cells were treated with 70% cold ethanol at 4°C overnight to increase cell membrane penetrability. Subsequently, 100 *μ*g/ml RNase (Keygentec, Nanjing, China) was used to treat cells at 37°C for 20 min. Following staining with 30 *μ*g/ml propidium iodide (Keygentec, Nanjing, China) for 30 min at room temperature in the dark, cell cycle was analyzed by Gallios Flow Cytometry (Beckman Coulter, USA). The flow cytometry results were evaluated using a Cell Quest kit (BD Biosciences, CA, USA).

### 2.7. Quantitative Reverse Transcription-PCR (RT-qPCR)

Total RNA was extracted using the TRIzol Reagent (Invitrogen, Carlsbad, CA, USA). RNA was reverse-transcribed to cDNA using the QuantiTect RT kit (Qiagen) following the manufacturer's instructions. Real-time PCR was performed using a System 7500 instrument (Applied Biosystems, Carlsbad, CA, USA) and the following PCR cycling protocol was used: 95°C for 5 min and then 40 cycles of 94°C for 15 s, 55°C for 20 s, and 72°C for 20 s, followed by 72°C for 7 min. The mRNA expression level of GAPDH was the endogenous control. The primer sequences for qPCR were as follows: SIRT7, forward 5′-ACGCCAAATACTTGGTCGTCT-3′ and reverse 5′-AGCACTAACGCTTCTCCCTTT-3′; GAPDH, forward 5′-CTCACCGGATGCACCAATGTT-3′ and reverse 5′-CGCGTTGCTCACAATGTTCAT-3′. Results were analyzed following the 2^-ΔΔCq^ calculation method [25].

### 2.8. Western Blotting

VSMCs proteins were extracted with RIPA lysis buffer (Beyotime, China) and incubated for 30 min on ice. Then the supernatants were collected after centrifugation. The concentrations were detected using a BCA protein assay kit (Bio-Rad, China). Equal amounts of proteins were loaded onto 10% SDS-polyacrylamide gels to separate various proteins which were transferred to PVDF membranes subsequently. Then these membranes were blocked with a buffer containing 10% non-fat milk in PBS for 2 h at room temperature, followed by incubation with primary antibodies overnight at 4°C, respectively. Then the membranes were incubated with the goat anti-rabbit horseradish peroxidase-conjugated IgG secondary antibodies (1:1,000; cat. no. A0208; Beyotime Institute of Biotechnology, Haimen, China) at room temperature for 1 h. Following washing three times with TBST, signals were detected using the enhanced chemiluminescence reagent (Millipore, Billerica, MA, USA) and the ImageJ software was provided to analyze the fold changes of protein levels. Anti-*α*-SMA (1:1000; cat. no. 19245S), anti-SIRT7 (1:1000; cat. no. 5360S), anti-*β*-catenin (1:1000; cat. no. 8480T), anti-cyclin D1 (1:1000; cat. no. 3300T), and anti-GAPDH (1:1000; cat. no. 5174S) antibodies were purchased from Cell Signaling Technology (Boston, MA, USA). Anti-Wnt (1:1000; cat. no. ab28472) antibody was the product of Abcam company (Cambridge, UK).

### 2.9. Statistical Analysis

All statistical analyses were conducted using SPSS 14.0 software (Chicago, IL). All experimental results were expressed as mean ± SD. Statistical comparisons were made by two-tailed Student's* t* test or one-way analysis of variance (ANOVA). P<0.05 was considered statistically significant.

## 3. Results

### 3.1. The Expression of SIRT7 Is Suppressed Highly in ox-LDL-Stimulated HAVSMCs

**Figure 1:**
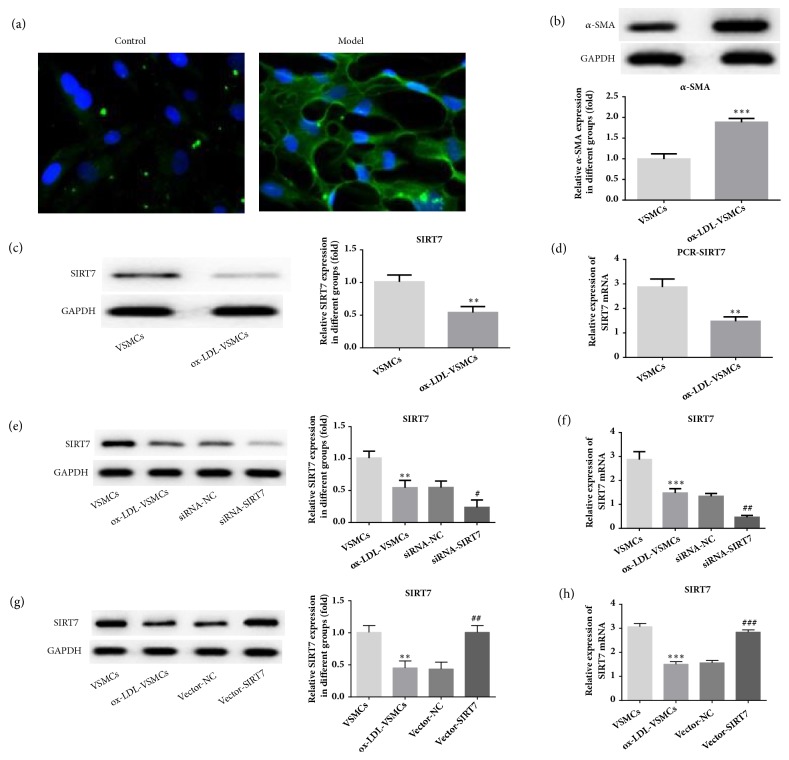
The *α*-SMA and SIRT7 expression in ox-LDL-treated HAVSMCs. (a) Immunofluorescence staining was used to detect *α*-SMA in HAVSMCs stimulated with ox-LDL. Nuclei were stained with DAPI (blue), 200× magnification. (b) The protein expression level of *α*-SMA was measured by Western blot. ^*∗∗∗*^P < 0.001 versus VSMCs. The protein level of SIRT7 was measured by Western blot (c) and RT-qPCR (d) in ox-LDL-treated HAVSMCs. ^*∗∗*^P < 0.01 versus VSMCs. The expression level of SIRT7 was measured by Western blot (e) and RT-qPCR (f) after silencing of SIRT7 in ox-LDL-treated HAVSMCs. ^*∗∗*^P < 0.01 and ^*∗∗∗*^P < 0.001 versus VSMCs; ^#^P < 0.05 and ^##^P < 0.01 versus siRNA-NC. The expression level of SIRT7 was measured by Western blot (g) and RT-qPCR (h) after overexpression of SIRT7 in ox-LDL-treated HAVSMCs. ^*∗∗*^P < 0.01 and ^*∗∗∗*^P < 0.001 versus VSMCs; ^##^P < 0.01 and ^###^P < 0.001 versus Vector-NC.

To determine whether the successful AS cells model was made, the immunofluorescence staining and protein analysis were employed in the present study. Our data indicated that treatment with ox-LDL led to a marked increase in the level of VSMC-specific marker gene *α*-SMA in immunofluorescence imaging [26], which was consistent with the *α*-SMA protein expression level (Figures 1(a) and 1(b)).

### 3.2. Silencing or Overexpression of SIRT7 Affects Cells Proliferation In ox-LDL-Stimulated HAVSMCs

**Figure 2:**
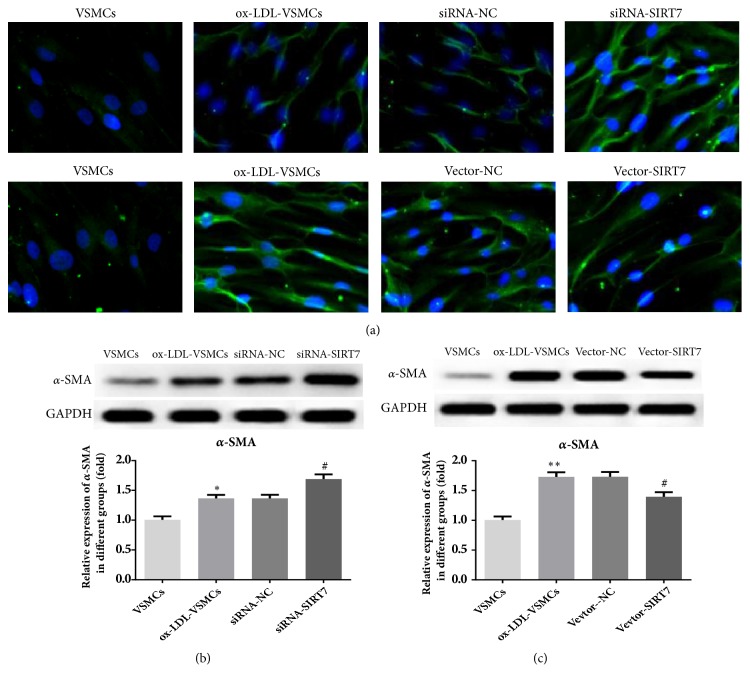
(a) The level of *α*-SMA after SIRT7 silencing or overexpression in ox-LDL-treated HAVSMCs was measured by immunofluorescence staining. Nuclei were stained with DAPI (blue), 200× magnification. (b) The protein expression level of *α*-SMA after SIRT7 silencing (b) or overexpression (c) in ox-LDL-treated HAVSMCs was assessed by Western blot. ^*∗*^P < 0.05 and ^*∗∗*^P < 0.01 versus VSMCs; ^#^P < 0.05 versus siRNA-NC or Vector-NC.

**Figure 3:**
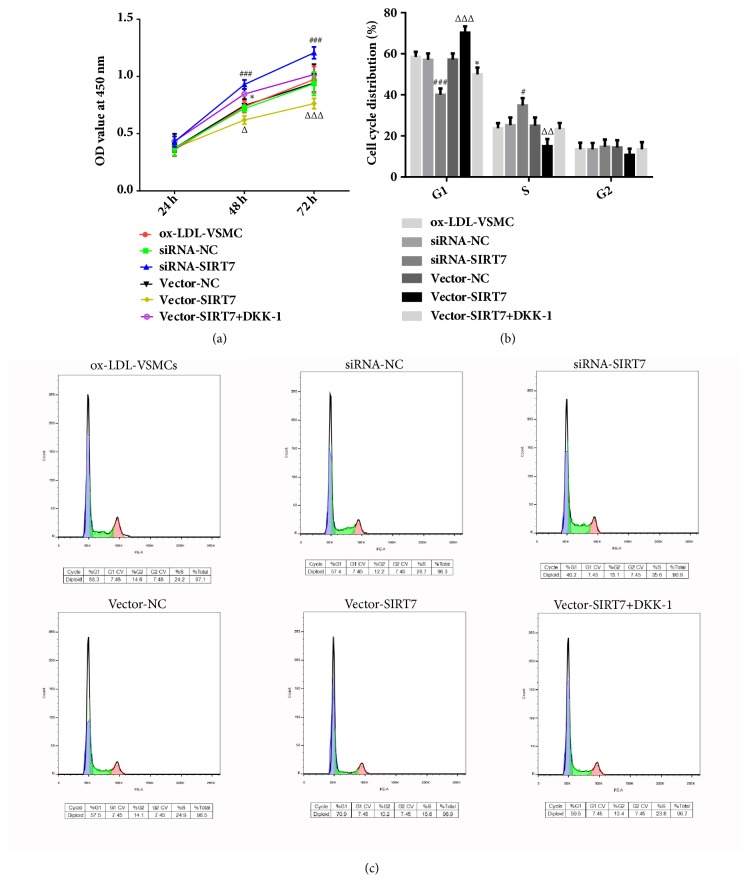
The effects of SIRT7 knockdown, overexpression, or treatment with DKK-1 on HAVSMCs proliferation stimulated by ox-LDL. (a) CCK-8 cell proliferation assay. ^###^P < 0.001 versus siRNA-NC; ^△^P < 0.05 and ^△△△^P < 0.001 versus Vector-NC; ^*∗*^P < 0.05 versus Vector-SIRT7. (b) Cell cycle distribution (%) in G1, S, and G2 phases. ^#^P < 0.05 and ^###^P < 0.001 versus siRNA-NC; ^△△^P < 0.01 and ^△△△^P < 0.001 versus Vector-NC; ^*∗*^P < 0.05 versus Vector-SIRT7. (c) Cell cycle analysis.

In our study, the results demonstrated that the protein and mRNA expression levels of SIRT7 were reduced after HAVSMCs underwent ox-LDL stimulation (Figures 1(c) and 1(d)). To test the effects of SIRT7 on cells proliferation, SIRT7 silencing or overexpression was made with siRNA or pcDNA3.1 plasmid successfully, which was shown from the decreasing or increasing protein and mRNA expressions of SIRT7 (Figures 1(e)–1(h)). At the same time, the expression of *α*-SMA was increased in siRNA-SIRT7 group. However, the level of *α*-SMA was decreased after SIRT7 overexpression (Figure 2). In addition, the CCK-8 assay suggested that SIRT7 knockdown promoted HAVSMCs proliferation induced by ox-LDL (Figure 3(a)), while overexpression of SIRT7 had opposite results. Furthermore, cell cycle analysis indicated that the percentage of cells at S phase in siRNA-SIRT7 group was significantly higher than that of siRNA-NC group, while G1 and G2 phases exhibited reverse results, whereas the SIRT7 overexpression exhibited a cycle arrest (Figures 3(b) and 3(c)). The results indicate that SIRT7 inhibition promotes cell proliferation, while SIRT7 overexpression impedes it in ox-LDL-stimulated HAVSMCs.

### 3.3. Silencing or Overexpression of SIRT7 Affects Cells Migration in ox-LDL-Stimulated HAVSMCs

**Figure 4:**
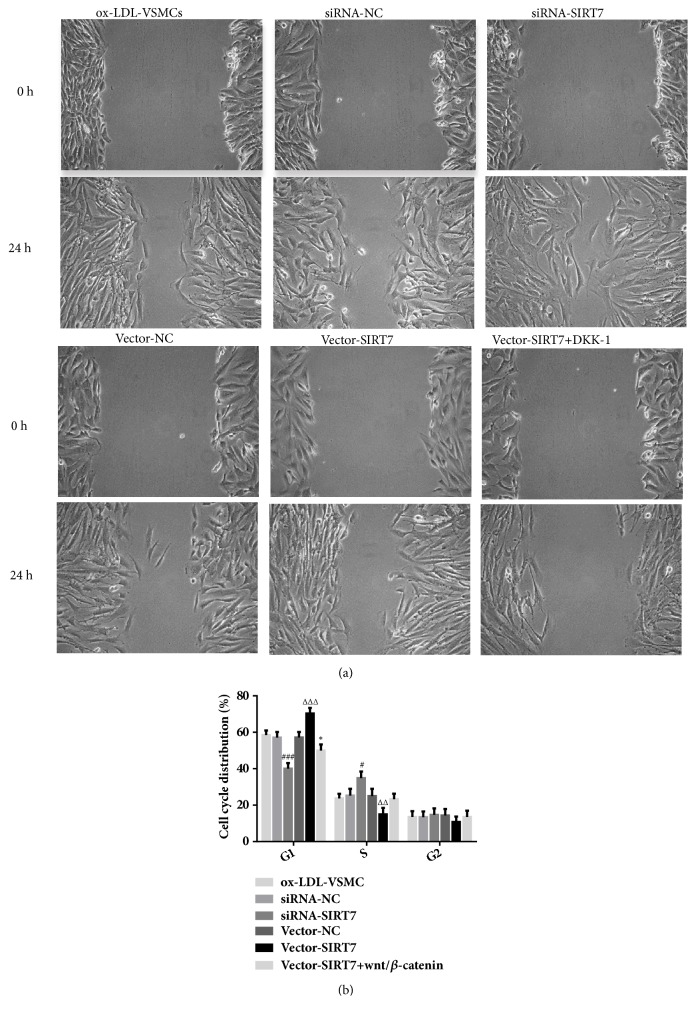
The effects of SIRT7 knockdown, overexpression, or treatment with DKK-1 on HAVSMCs migration stimulated by ox-LDL. (a) Images of the migration of HAVSMCs at 0 and 24 h (4× magnification). (b) The wound closure of ox-LDL treatment groups at 0 and 24 h. ^###^P < 0.001 versus siRNA-NC; ^△△△^P < 0.001 versus Vector-NC; ^*∗∗*^P < 0.01 versus Vector-SIRT7.

We monitored cells migration by wound-healing assay. From the results, we found that the relative distance was decreased in SIRT7 knockdown group compared with that in knockdown negative control group at the time of 24 h after scratch (Figures 4(a) and 4(b)). Importantly, the relative distance was increased after transfection with pcDNA3.1 plasmid. From these results, we find that SIRT7 silencing accelerates cell migration, whereas SIRT7 overexpression retards it in ox-LDL-stimulated HAVSMCs.

### 3.4. SIRT7 Regulates Cells Proliferation and Migration via Wnt/*β*-Catenin Signaling Pathway

**Figure 5:**
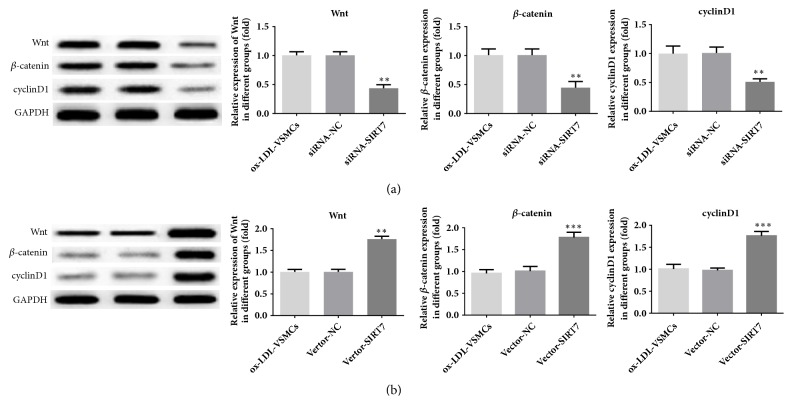
The protein expression levels of Wnt, *β*-catenin, and cyclin D1. (a) The protein expression levels of Wnt, *β*-catenin, and cyclin D1 in SIRT7 knockdown HAVSMCs. ^*∗∗*^P < 0.01 and ^*∗∗∗*^P < 0.001 versus ox-LDL-VSMC; ^##^P < 0.01 and ^###^P < 0.001 versus siRNA-NC. (b) The protein expression levels of Wnt, *β*-catenin, and cyclin D1 in SIRT7 overexpression on HAVSMCs. ^*∗∗∗*^P < 0.001 versus ox-LDL-VSMC; ^###^P < 0.001 versus Vector-NC.

To further explore the molecular mechanism underlying the SIRT7-mediated protective effects on ox-LDL-induced proliferation and migration of HAVSMCs, the following study investigated the relative variation in Wnt/*β*-catenin signaling. After treatment with ox-LDL, the protein expression levels of Wnt, *β*-catenin, and cyclin D1 were decreased extremely in SIRT7 knockdown HAVSMCs (Figure 5(a)); however, they were increased obviously in SIRT7 overexpression HAVSMCs (Figure 5(b)). Furthermore, DKK-1, a secretory glycoprotein, was used to block the Wnt/*β*-catenin signaling pathway in our study. The results indicated that the abilities of cells proliferation and migration were augmented after DKK-1 was employed in SIRT7 overexpression HAVSMCs, which were exhibited in Figures 3 and 4. These results uncover that SIRT7 regulates cells proliferation and migration via Wnt/*β*-catenin signaling pathway.

## 4. Discussion

It is well known that AS is a chronic degenerative disease and has become a predominant factor of a plenty of cardiovascular diseases, which has high morbidity and mortality. However, the specific pathogenesis mechanisms of AS are still not fully elucidated. In this study, we found that SIRT7 regulates the VSMCs proliferation and migration induced by ox-LDL via Wnt/*β*-catenin signaling pathway.

Aberrantly proliferation and migration of VSMCs play a considerable role in the progression of AS [27]. It has been well documented that ox-LDL exerts a promotion effect on cellular proliferation and migration [28]. Evidence indicates that SITR7 as an important cellular regulator plays critical roles in cell proliferation, migration, survival, and protein synthesis [11–13]. Furthermore, SIRT7 possesses antiproliferative effects in the control of cellular growth and proliferation [12, 29], which suggests that SIRT7 may inhibit the development procession of AS. Decreased expression level of SIRT7 was detected in ox-LDL-treated HAVSMCs in our study. Additionally, silencing of SIRT7 by siRNA considerably accelerated cellular proliferation, coupled with the increasing percentage of VSMCs at S phase and decreasing that of G1 and G2 phases detected by cell cycle analysis. At the same time, cellular migration was expedited assessed by the wound-healing assay. Moreover, overexpression of SIRT7 exhibited an inhibition effect on HAVSMCs proliferation and migration in our study. To the best of our knowledge, the results indicate that SIRT7 plays a protective role in regulating cellular proliferation and migration in ox-LDL-induced HAVSMCs.

The Wnt/*β*-catenin signaling is an evolutionarily conserved pathway and activation of this signaling regulates the expression of downstream target genes including cyclin D1, subsequently resulting in many variations of pathophysiological behaviors, such as cell differentiation, proliferation, migration, and polarity [30, 31]. Accumulating evidence indicated that Wnt/*β*-catenin signaling has been shown to be involved in SMA and calponin expression which are marker genes during the progression of AS [32, 33]. In addition, it has been reported that SIRT7 is essential for osteogenic differentiation of bone marrow stem cells, partly by activation of the Wnt/*β*-catenin signaling pathway [18]. To investigate the potential mechanisms of SIRT7-mediated protective effects on ox-LDL-induced proliferation and migration of HAVSMCs, Wnt/*β*-catenin signaling intrigues our interest because of its crucial role in regulating essential aspects of cell proliferation and migration [34, 35]. The present study aimed to evaluate whether Wnt/*β*-catenin signaling was affected by the knockdown or overexpression of SIRT7 in HAVSMCs. In our present research, we found that the expression levels of *β*-catenin and cyclin D1 were notably decreased in SIRT7 silencing HAVSMCs, while an increased result was detected in SIRT7 overexpression condition. Taken together, these findings indicated that there was a regulatory relationship between SIRT7 and Wnt/*β*-catenin signaling in HAVSMCs.

To further elucidate the underlying regulation between SIRT7 and Wnt/*β*-catenin signaling, DKK-1, a classical inhibitor of canonical Wnt/*β*-catenin signaling, was employed in our study [36]. The results indicated that the levels of HAVSMCs proliferation and migration induced by ox-LDL were remarkably decreased assessed by CKK-8, cytometric analysis, and wound-healing assay, respectively, which verified our hypothesis and further suggested that SIRT7 regulates the VSMCs proliferation and migration in AS via Wnt/*β*-catenin signaling pathway.

## 5. Conclusion

According to our results, the present study demonstrated that SIRT7 was significantly downregulated in ox-LDL-stimulated HAVSMCs. SIRT7 knockdown notably promoted cell proliferation and migration and downregulated the expression of *β*-catenin and cyclin D1, while SIRT7 overexpression had reverse results. These findings indicated that SIRT7 plays a protective role in regulating proliferation and migration of AS via Wnt/*β*-catenin signaling pathways.

## Data Availability

The data used to support the findings of this study are included within the article.
