# Erratum to “SIRT7 Regulates the Vascular Smooth Muscle Cells Proliferation and Migration via Wnt/β-Catenin Signaling Pathway”

**DOI:** 10.1155/2019/8014381

**Published:** 2019-11-04

**Authors:** BioMed Research International

In the article titled “SIRT7 Regulates the Vascular Smooth Muscle Cells Proliferation and Migration via Wnt/*β*-Catenin Signaling Pathway” [1], there was an error that occurred during the production stage, as Figure 4(b) was wrongly replaced with a copy of Figure 3(b). Therefore, Figures 3(b) and 4(b) are identical. However, Figure 1 should be corrected as follows:

The authors could not be reached.

## Figures and Tables

**Figure 1:**
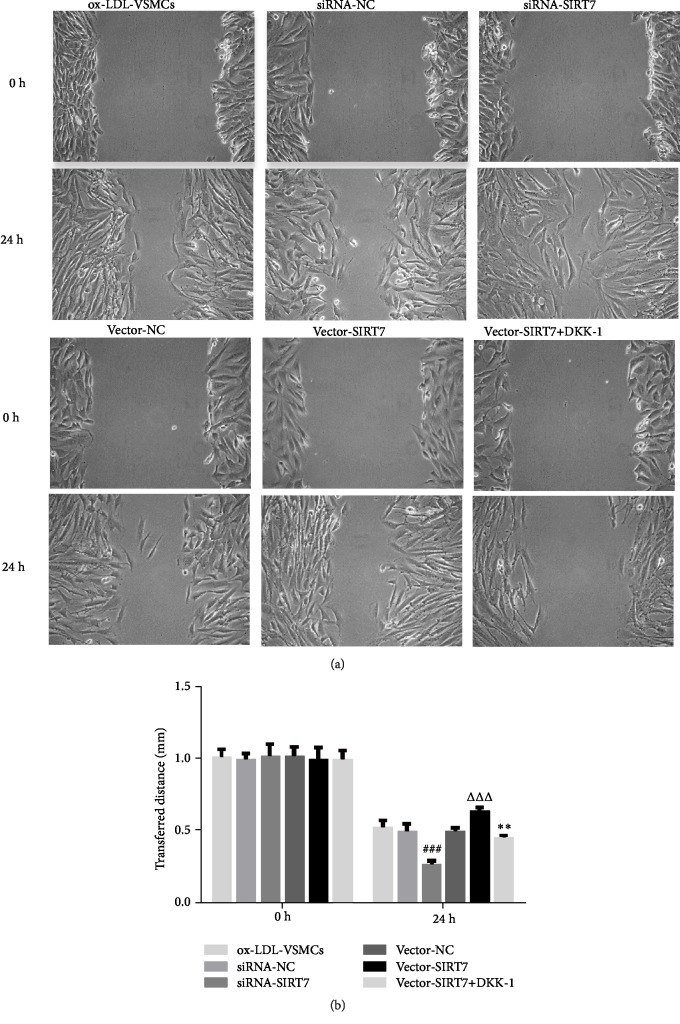
The effects of SIRT7 knockdown, overexpression, or treatment with DKK-1 on HAVSMCs migration stimulated by ox-LDL. (a) Images of the migration of HAVSMCs at 0 and 24 h (4× magnification). (b) The wound closure of ox-LDL treatment groups at 0 and 24 h. ^###^P < 0.001 versus siRNA-NC; ^△△△^P < 0.001 versus Vector-NC; ^*∗∗*^P < 0.01 versus Vector-SIRT7.
